# Fluoride Removal Using Nanofiltration-Ranged Polyamide Thin-Film Nanocomposite Membrane Incorporated Titanium Oxide Nanosheets

**DOI:** 10.3390/nano14080731

**Published:** 2024-04-22

**Authors:** Fekri Abdulraqeb Ahmed Ali, Javed Alam, Saif M. H. Qaid, Arun Kumar Shukla, Ahmed S. Al-Fatesh, Ahmad M. Alghamdi, Farid Fadhillah, Ahmed I. Osman, Mansour Alhoshan

**Affiliations:** 1 Chemical Engineering Department, College of Engineering, Imam Mohammad Ibn Saud Islamic University (IMSIU), Riyadh 11432, Saudi Arabia; feaali@imamu.edu.sa (F.A.A.A.); amsalghamdi@imamu.edu.sa (A.M.A.); fffadhillah@imamu.edu.sa (F.F.); 2 King Abdullah Institute for Nanotechnology, King Saud University, P.O. Box 2455, Riyadh 11451, Saudi Arabia; ashukla@ksu.edu.sa; 3 Department of Physics & Astronomy, College of Sciences, King Saud University, P.O. Box 2455, Riyadh 11451, Saudi Arabia; sqaid@ksu.edu.sa; 4 Chemical Engineering Department, College of Engineering, King Saud University, P.O. Box 2455, Riyadh 11451, Saudi Arabia; aalfatesh@ksu.edu.sa; 5 School of Chemistry and Chemical Engineering, Queen’s University Belfast, Belfast BT9 5AG, Northern Ireland, UK

**Keywords:** defluoridation, thin film nanocomposite membrane, titanium oxide nanosheets, Donnan-steric-pore-model-dielectric-exclusion

## Abstract

Drinking water defluoridation has attracted significant attention in the scientific community, from which membrane technology, by exploring thin film nanocomposite (TFN) membranes, has demonstrated a great potential for treating fluoride-contaminated water. This study investigates the development of a TFN membrane by integrating titanium oxide nanosheets (TiO_2_ NSs) into the polyamide (PA) layer using interfacial polymerization. The characterization results suggest that successfully incorporating TiO_2_ NSs into the PA layer of the TFN membrane led to a surface with a high negative charge, hydrophilic properties, and a smooth surface at the nanoscale. The TFN membrane, containing 80 ppm of TiO_2_ NSs, demonstrated a notably high fluoride rejection rate of 98%. The Donnan-steric-pore-model-dielectric-exclusion model was employed to analyze the effect of embedding TiO_2_ NSs into the PA layer of TFN on membrane properties, including charge density (X_d_), the pore radius (r_p_), and pore dielectric constant (ε_p_). The results indicated that embedding TiO_2_ NSs increased X_d_ and decreased the εp by less than the TFC membrane without significantly affecting the r_p_. The resulting TFN membrane demonstrates promising potential for application in water treatment systems, providing an effective and sustainable solution for fluoride remediation in drinking water.

## 1. Introduction

The contamination of drinking water with fluoride introduces health hazards, for instance, dental and skeletal fluorosis [1,2]. The levels of fluoride in natural water reservoirs increase due to geogenic factors, like the dissolution of rocks and sediments containing fluorine-based minerals, as well as anthropogenic factors, primarily linked to the usage of pesticides and industrial operations [3]. As a result, the issue of fluoride contamination in drinking water has emerged as a significant concern in various nations, including Mexico, Argentina, the United States, countries in the Middle East, India, and China [4].

Defluoridation poses a significant challenge in reducing fluoride concentration to meet acceptable standards set by the World Health Organization (WHO) for potable water, which is below 1.5 mg/L [5]. Numerous technologies have been utilized for the eradication of fluoride from water, encompassing adsorption [6,7], membrane separation [8,9], ion exchange [10,11], and precipitation–coagulation [12]. Within these technologies, membrane-based processes have garnered considerable interest in removing fluoride from water in recent times owing to their commendable efficiency and dependability. Nanofiltration (NF), a highly efficient membrane process, presents a commendable equilibrium between permeability, selectivity, and energy prerequisites [13]. Compared to reverse osmosis (RO) membranes, NF has a lower ion rejection because the selectivity membranes rely on a combination of steric and charge interactions [9]. For this reason, the selectivity of NF is enhanced by incorporating nanoparticle materials, which improve morphological features, like roughness, thickness, and pore size, as well as surface properties, such as charge and hydrophilicity [14]. Recently, TiO_2_ nanoparticles have garnered considerable interest due to their distinct characteristics, notably their exceptional ability to combat bacterial growth, high hydrophilicity, strong chemical stability [15], and low toxicity [16]. The nanostructure of TiO_2_, specifically TiO_2_ NSs, in a two-dimensional (2D) form provides an opportunity to bring out more intriguing characteristics than from its one-dimensional counterpart. These include a significantly increased surface area, an ultra-thin thickness, and the occurrence of quantum confinement [17]. While TiO_2_ NSs have found extensive use in various applications, their utilization in improving TFC membrane properties represents a relatively new approach, with limited research conducted in this area. For instance, Shuangmei Xue et al. successfully fabricated an interlayer-enhanced TFC membrane using 2D titania NSs, achieving improved separation performances with high water permeability [18]. Similarly, Nor Akalili Ahmad et al. employed a layer-by-layer (LbL) assembly technique to deposit a thin layer of titania NSs on the PA TFC membrane, achieving significantly enhanced water permeability of 0.8 L·m^−2^·h^−1^·bar^−1^ (60% improvement) alongside high sodium chloride (NaCl) rejection of 98.45% [19].

The novelty of this investigation lies in the incorporation of TiO_2_ NSs into the PA layer of TFN to enhance the efficacy of fluoride removal from water. The incorporation of TiO_2_ NSs led to enhancements in the properties of the TFN membranes, such as surface charge, smoothness, and hydrophilicity, ultimately resulting in improved separation capabilities. The TFN membrane was fabricated by carrying out interfacial polymerization (IP), wherein a TiO_2_ NSs-dispersed m-phenylenediamine (MPD) aqueous solution reacted with an organic solution of trimesoyl chloride (TMC) on a top porous polysulfone (PSF) support, resulting in the formation of a thin layer of PA. The separation efficiency of the TiO_2_ NS-incorporated TFN membranes was assessed by investigating the rejection of fluoride and the flux of water. The experimental data pertaining to fluoride rejections were analyzed using the DSPM-DE Model to investigate the influence of incorporating TiO_2_ NSs into the thin PA layer on various membrane properties, including X_d_, r_p_, and ε_p_.

## 2. Experimental

### 2.1. Materials and Chemicals

BASF, located in Germany, graciously supplied the PSF polymer (P-3500 LCD, Mwt.: 75–81 × 10^3^ gm/mol). Polyethylene glycol (PEG 600) and TMC monomer (with a purity level of 98%) were procured from Merck (Darmstadt, Germany). Sigma-Aldrich (Kenilworth, NJ, USA) served as the source for the MPD monomer (purity level of 99%) and sodium dodecyl sulfate (SDS). Essential chemicals, including NMP (with a purity level of 99%), hexane (C_6_H_14_, purity level of 99%), trimethylamine (TEA, purity level of 99%), and inorganic materials such as NaCl, Na_2_SO_4_, MgSO_4_, and NaF salts (with a purity level of 98%), were procured from Oxford Lab Chem (Palghar, Maharashtra, India). All experimental procedures were carried out using deionized water (Milli-Q), having a resistivity of 18.2 Mcm.

The TiO_2_ NSs utilized in this study were prepared, ensuring exposed {001} facets, following a previously reported method by Ghaithan et al. (2015) [20,21]. The morphologies of the TiO_2_ NSs were thoroughly examined using transmission electron microscopy (TEM, JEOL, Japan). 

### 2.2. Fabrication of Polysulfone Support Layer

Porous support layers of the TFN membrane were fabricated via a phase inversion technique. Initially, the solution was prepared by dissolving 17 wt% of PSF polymer and 15 wt% of PEG 600 (the pore-forming agent) in NMP solvent at 70 °C with continuous stirring until complete dissolution. Subsequently, the homogeneous polymer solution was poured onto a plate made of glass and cast using a knife film applicator (DeltaE Srl, Rende, Italy). Then, immediately, the cast films were immersed in a coagulation bath at 25 °C and left for 24 h to ensure that the phase inversion process was completed. 

### 2.3. Preparation of Thin Film Nanocomposite Membrane

**Figure 1:**
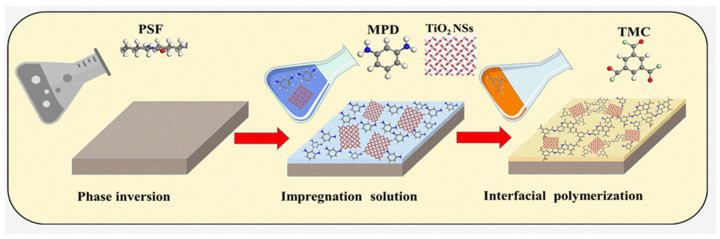
Schematic illustration of TFN membrane fabrication.

**Figure 2:**
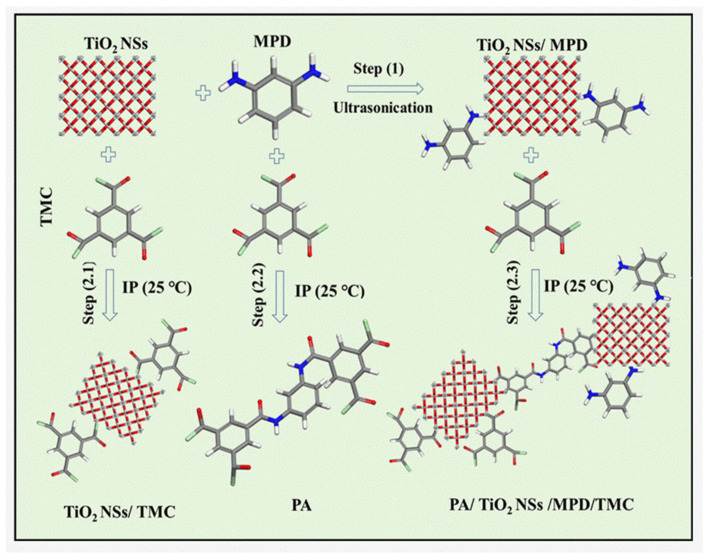
Schematic illustration of interactions of TiO_2_ NSs with TMC and MPD.

The TFC and TFN membranes were synthesized via an IP reaction, as shown in the fabrication scheme in Figure 1. Initially, a solution of MPD was prepared by dissolving 1 wt./v% MPD, 0.2 wt./v% SDS, and 1 wt./v% TEA in DI water [22,23]. Various concentrations of TiO_2_ NSs (20, 40, and 80 ppm) were uniformly dispersed in the solution containing MPD monomer using a digital sonifier (Branson Ultrasonics Corporation, Brookfield, CT, USA) through ultrasound sonication for 20 min to form MPD/TiO_2_ aqueous solutions. Next, 50 mL of the MPD/TiO_2_ aqueous solution was carefully poured onto the prepared PSF support layer. This amalgamation was given 10 min to enable the MPD/TiO_2_ solution to permeate into the PSF support layer. The excess aqueous solution containing MPD/TiO_2_ was drained off, and any remaining solution on the surface was removed utilizing a rubber roller. Subsequently, 0.1 wt./v% TMC was dissolved in 30 mL of n-hexane and poured onto the MPD/TiO_2_-impregnated PSF support surface to create a thin layer of PA through a reaction with the MPD monomer, lasting 60 s. The scheme illustrating the potential reaction mechanism can be observed in Figure 2. The fabricated TFN membranes were rinsed with hexane to remove unreacted solution and cured in an air oven at 80 °C for 5 min. Then, the resultant membrane was washed with DI water and rinsed in DI water. The TFN membranes were placed in a refrigerator adjusted to a temperature of 5 °C for storage. The same procedure was explored to fabricate a TFC membrane without the inclusion of TiO_2_.

### 2.4. Membrane Characterizations 

The surface morphology of the fabricated TFN membranes was examined using a field-emission scanning electron microscope (FE-SEM, JEOL, Tokyo, Japan). The membrane samples were affixed to holders using carbon tape for the SEM study. These holders were subsequently inserted into a platinum sputtering machine to apply a platinum coating onto the samples. The surface morphology of the platinum-coated membrane samples was then analyzed using SEM under high vacuum conditions. The roughness was assessed using an atomic force microscope (AFM, Veeco NanoScope V Multi-Mode software, version 6.13). The impact of TiO_2_ NSs on the membrane surface charge was investigated using a SurPASS electro-kinetic analyzer (Anton-Paar KG, Graz, Austria) to measure the zeta potential. All membrane ζ potential measurements were performed using a 1 mM KCl electrolyte solution at ambient temperature. The pH of the KCl solution was adjusted from 2.5 to 9.8 using solutions of 0.05 M HCl and 0.05 M NaOH in an automatic titration unit. 

A water contact angle measuring system (Atension, MAC 200, Ultrecht, The Netherlands) was utilized to characterize the surface hydrophilicity of the membranes using the sessile drop technique. The surface elemental compositions of TFC and TFN membranes were quantified using an X-ray photoelectron spectroscopy (XPS, JEOL, JPS-9030, Tokyo, Japan) equipped with a Mg Kα (1253.6 eV) X-ray source. The spectrometer operated at 10 mA and 12 kV in ultrahigh vacuum conditions (<10^–7^ Pa). Attenuated Total Reflectance-Fourier Transform Infrared (ATR-FTIR) spectroscopy (Thermo Scientific, Winsford, UK) was utilized to quantify the functional groups of TFC and TFN membranes within the spectral range of 500–2000 cm^−1^. 

### 2.5. TFN Membranes’ Performance Analysis

Permeate flux and salt rejection were measured using a cross-flow cell system powered by a high-pressure pump from WASHGUARD^®^ SST^™^ (MODEL NO. C6T17FK83). Four cells were used in the filtration setup, each with an effective area of 42 cm^2^ and sourced from Sterlitech Corporation (Kent, WA, USA). This allowed for the simultaneous evaluation of multiple membranes under identical conditions. Initially, experimental DI water was utilized to condition fresh membranes at 6 bar until their flux stabilized. The measurement of water flow across TFN membranes was conducted. Subsequently, water flux was calculated by employing Equation (1) under different Transmembrane Pressures (TMPs) denoted as P:

(1) $$J_{m}=\frac{Q_{m}}{A_{m}∆t}$$

where J_m_ represents the permeate flux (L m^−2^ h^−1^), Q_m_ is expressed as the water-permeated volume (L), Am is the effective area (m^2^), and ∆t is the permeation duration (h). 

The evaluation of salt rejection in TFN membranes was conducted by employing a solution consisting of a single solute, specifically NaCl (2000 ppm), Na_2_SO_4_ (1000 ppm), and MgSO_4_ (1000 ppm), this evaluation under an applied pressure of 5 bar, a flow rate of 1.4 L/min, and cross-flow velocity of 0.265 m/s. The salt concentration in the permeate was determined utilizing a conductivity meter (DeltaOhm HD 2156.1). For fluoride rejection experiments, solutions with varying fluoride concentrations (ranging from 5 to 500 mg/L), pressures (2, 3, 4, and 5), and pH values (4 to 10) were utilized. The permeate and concentrate streams were recirculated into the feed tank to maintain a constant concentration. Fluoride concentration in the permeate was measured using a pH/fluoride Meter (WS 100 Model, APERA, Columbus, OH, USA), initially calibrated using standard fluoride samples. The fluoride removal percentage was calculated using Equation (2):

(2) $$R\%=1-\frac{C_{P}}{C_{F}}\times 100$$

where $C_{P}$ and $C_{F}$ represent fluoride concentration in the feed and permeate, respectively.

## 3. Theoretical Modelling Background

### 3.1. Donnan Steric Pore Model with the Dielectric Exclusion

The transport of electrolytes through membranes is described using a one-dimensional Donnan steric pore model with dielectric exclusion (DSPM-DE). This model expands upon the Nernst–Planck equation and incorporates three significant components defining the transportation of electrolytes across the thin PA active layer of NF/RO membranes. These components encompass diffusion, convection, and electromigration. The expanded Nernst–Planck equation, with other supporting equations, is shown in Table 1.

### 3.2. The Supply–Demand-Based Optimization Algorithm

The Supply–Demand-Based Optimization (SDO) algorithm was employed to fit the experimental data of fluoride rejections with the DSPM-DE Model. This fitting aimed to evaluate the influence of TiO_2_ NSs incorporated into the PA layer on the TFN membrane parameters, specifically X_d_, r_p_, and ε_p_. The objective of the fitting procedure was to minimize the total sum of squared deviations (SSE) between the observed rejection (R_exp_) obtained through experimentation and the rejection estimated by the DSPM-DE Model, as illustrated in Equation (14).

(14) $$minSSE=\sum_{i=1}^{N}{\left(R_{exp}-R_{mod}\right)}^{2}$$

N represents the number of data points in the experimental rejection dataset. The SSE was minimized to obtain the most suitable parameter values (i.e., r_p_, X_d_, and ε_p_) for the fabricated TFN membranes, ensuring a precise fit between experimental and model-predicted NaF rejections.

### 3.3. Procedure to Estimate the Parameters 

**Figure 3:**
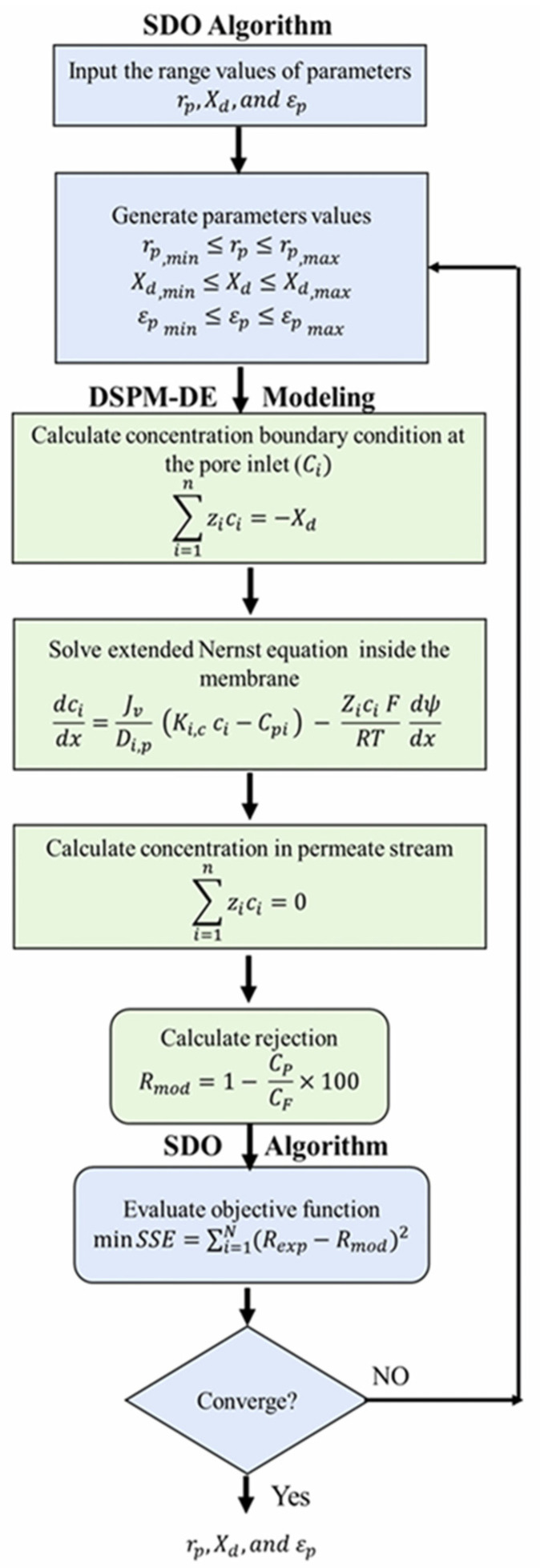
Diagram illustrating the sequential process of resolving numerical values in the DSMP-DE Model with the SDO algorithm.

The data on fluoride rejection obtained from the experiment was analyzed using the DSPM-DE Model with the aid of the SDO algorithm. This facilitated the determination of the optimal values for the membrane parameters. A thorough fluoride rejection study was conducted, encompassing various fluoride concentrations (ranging from 5 to 500 mg/L) and pH levels (ranging from 4 to 9.5) within the feed solution. The solution’s pH was carefully adjusted by utilizing HCl and NaOH solutions. In contrast, the measurement of its pH was aided by a Multimeter (Denver Instruments, Arvada, CO, USA, Model 250) equipped with a pH/ATS electrode. The fitting process, which entailed utilizing the DSPM-DE Model and the DSO algorithm, was executed on a 64-bit computer operating on the Windows 10 platform. This computer, equipped with an Intel^®^ Core i7-10870H CPU running at a frequency of 2.20 GHz, boasting a substantial 16 M.B. of RAM.MATLAB-R2019a (version 9.6), was the chosen platform for executing this fitting procedure, ensuring computational accuracy and efficiency. The overall strategy of utilizing the SDO algorithm to predict membrane properties by fitting experimental data with the SPDM-DE Model is outlined in the flowchart depicted in Figure 3.

## 4. Results and Discussion

### 4.1. Membrane Characterization 

#### 4.1.1. Surface Morphology 

**Figure 4:**
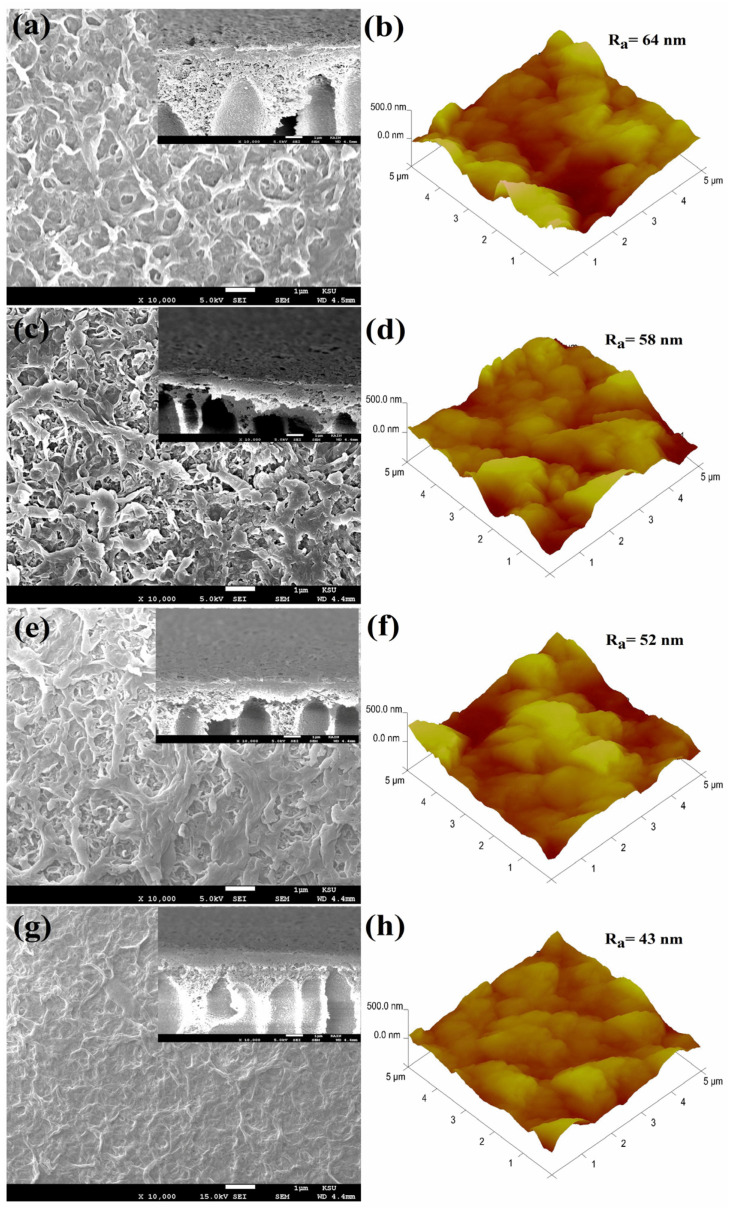
FE-SEM and AFM images: (**a**,**b**) TFC, (**c**,**d**) TFN-20 ppm, (**e**,**f**) TFN-40 ppm, and (**g**,**h**) TFN-80 ppm.

The nodular structures observed in the FE-SEM images of the TFC and TFN membranes, depicted in Figure 4, are characteristic features resulting from the IP process [26,27]. Figure 4a,c,e,g provide additional visual evidence that the surfaces of TFN membranes exhibit a denser configuration characterized by a leaflike morphology, in contrast to the nodular surface of the TFC membrane depicted in Figure 4a. The transformation above can be ascribed to the incorporation of TiO_2_ NSs in the IP reaction, exerting an effect on the degree of crosslinking within the PA layer. As a result, this phenomenon leads to a decrease in the nodular structure of the surface and the formation of a denser layer featuring a morphology of leaflike structure [28]. The TiO_2_ NSs, incorporated within the PA layer, underwent a physical interaction with the MPD monomer and TMC monomer during the IP reaction. As shown in Figure 4b,d,f,h, incorporating TiO_2_ into the thin PA layers reduced surface roughness for TFN membranes compared to pristine TFC membranes (Figure 4b). The reduction in surface roughness observed in the TFN membrane may be attributed to TiO_2_ NSs during the IP process. This presence is believed to enhance the formation of leaf-like structures on the thin PA layers’ surfaces, leading to a smoother surface. The obtained outcomes are consistent with the findings depicted in the FE-SEM images. These images reveal that TiO_2_ NSs impede the development of nodular structures [29,30].

#### 4.1.2. Interaction between TiO_2_ NSs and Polymer

**Figure 5:**
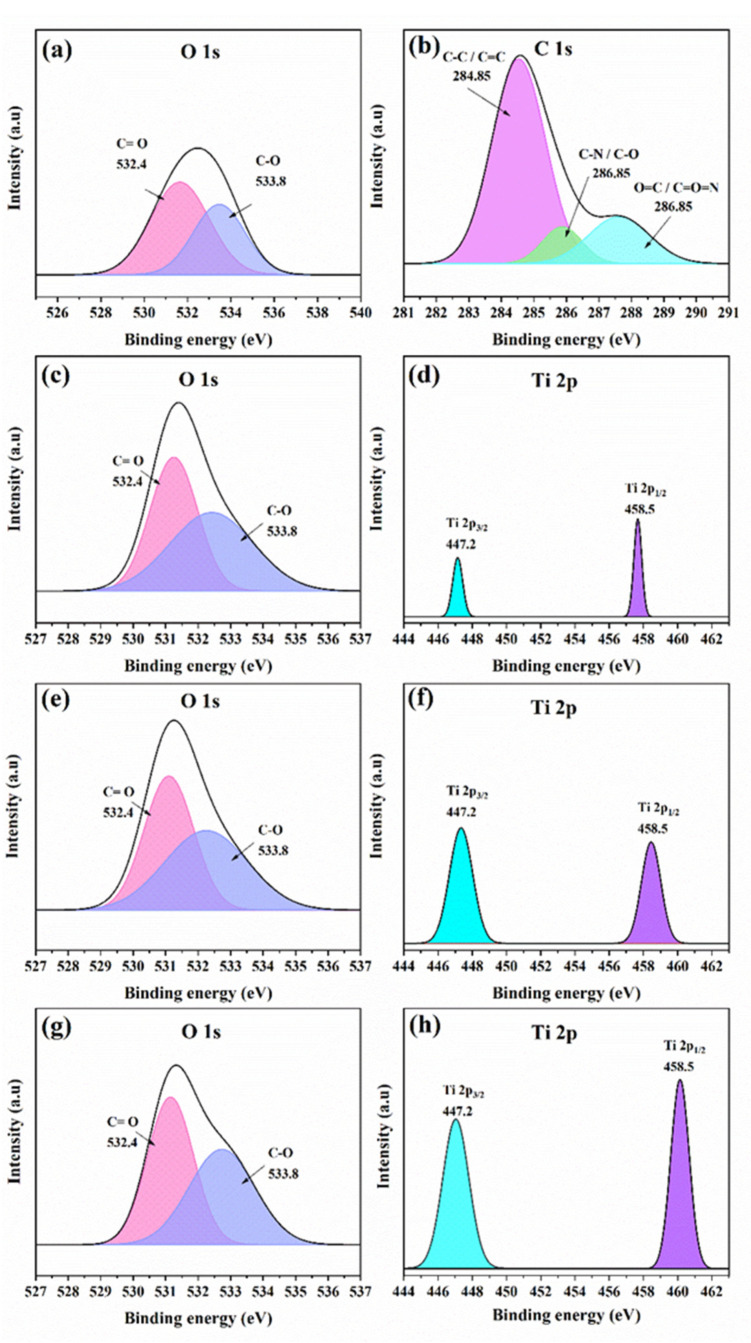
O1s X-ray photoelectron spectra of the (**a**) TFC, (**c**) TFN 20 ppm, (**e**) TFN 40 ppm, and (**g**) TFN 80 ppm membranes, C 1s X-ray photoelectron spectra of the TFC membranes (**b**), and Ti 2p X-ray photoelectron spectra of (**d**) TFN 20 ppm, (**f**) TFN 40 ppm, and (**h**) TFN 80 ppm membranes.

XPS was utilized to analyze the elemental composition and varieties of atomic bonds on the surface of the PA layer of the TFN membrane, thereby rendering valuable elucidations concerning the existence of the TiO_2_ NSs incorporated into the PA layer. The spectrum of the O 1s, depicted in Figure 5a, demonstrates a discernible peak at a vicinity of 532.4 eV, signifying the presence of C=O bonds. Consequently, it implies the participation of carbonyl groups in the composition of the surface. In addition, another peak at approximately 533.8 eV is ascribed to the presence of C–O bonds [31], thereby providing valuable insights into the composition and chemical interactions involving oxygen [32]. The spectrum of the C 1s displays three distinct peaks, as illustrated in Figure 5b. At approximately 284.85 eV, the initial peak is linked to bonds involving C–C and C=C. The second peak, around 286.85 eV, is attributed to bonds involving C–O and C=N [33,34]. The third peak, observed at approximately 287.81 eV, corresponds to bonds involving O=C and O=C=N [35].

The titanium spectrum (Ti 2p) peaks at about 447.2 eV, ascribed to Ti 2p_3/2_. This observation offers a valuable understanding of titanium species’ existence on the membrane’s surface. Furthermore, another evident peak observed at 458.5 eV can be attributed to the presence of Ti 2p_1/2_, thereby confirming the existence of the titanium species and their respective electronic states [36,37]. Figure 5f,h demonstrate a clear relationship where an increase in the concentration of incorporated TiO_2_ NSs into the PA layer results in a proportional increase in the intensity of the Ti 2p_3/2_ and Ti 2p_1/2_ peaks. This signifies a direct correlation between the amount of TiO_2_ NSs incorporated within the TFN membrane and the concentration of TiO_2_ NSs in the MPD solution. Further, it is noteworthy that Figure 5c,e,g exhibit an increase in the intensity of oxygen peaks after incorporating TiO_2_ NSs into the PA layer. The enhanced peaks displayed in the O 1s spectrum provide compelling evidence of an increase in the oxygen content closely linked to the amount of TiO_2_ NSs incorporated into the PA layer.

**Figure 6:**
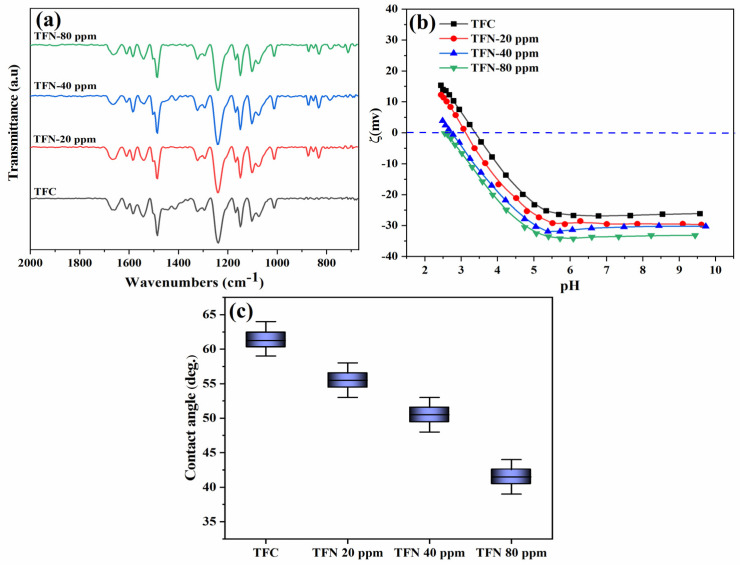
(**a**) ATR-FTIR spectrum, (**b**) Zeta potential results, and (**c**) water contact angles.

Figure 6a displays the ATR-FTIR spectra of the fabricated TFN membranes containing different TiO_2_ NSs incorporated within the PA layer. The ATR-FTIR dip probe can penetrate the membrane surface to approximately 0.45–0.55 µm depth. The probe can uncover peaks linked to the polysulfone substrate within the I.R. spectra through this penetration. In Figure 6a, peaks at 1238, 1295, and 1321 cm^−1^ correspond to symmetric stretching of the C-O-C bond, stretching of the S-O bond, and symmetric stretching of the C-SO_2_-C bond, respectively. In addition, the bending and rocking of the aliphatic C-C and aromatic C-H bonds within the polysulfone polymer chain contribute significantly to the responsible vibrational bands at 873, 1080, and 1014 cm^−1^ [38,39]. However, the spectrum of TFN membranes exhibits notable peaks representing amide groups that have been synthesized through interfacial polymerization. These peaks occur at 1100, 1583, and 1659 cm^–1^ and can be attributed to the bending vibrations of N–H, the stretching of C–N (amide), and the stretching of C=O (carboxylic), respectively [40,41]. The I.R. spectra of TFN membranes with incorporated TiO_2_ NSs exhibit a remarkable similarity to those of TFC membranes, suggesting that the interfacial polymerization occurred in TiO_2_ NSs. The 640 and 800 cm^−1^ peaks in the I.R. spectra can be attributed to the stretching of the Ti–O band, as reported in previous studies [42,43]. This observation shows the effective integration of TiO_2_ NSs within a thin PA layer.

#### 4.1.3. Surface Properties

##### Zeta Potential

The alterations in the surface charge of the TFN membranes at different pH levels are depicted in Figure 6b. The results obtained from the zeta potential analysis reveal that the TFN membranes’ surface charge became more negatively charged with the increasing amount of TiO_2_ NSs embedding into the active PA layers. As depicted in Figure 6b, the surface charge of the pristine membrane at pH 7 was −26 mV whereas, for the TFN membrane containing 80 ppm TiO_2_ NSs, it was −35 mV. This phenomenon can be attributed to the incorporation of hydrolyzed TiO_2_ into the PA layer, resulting in the formation of the negatively charged hydroxide functional group [44,45].

##### Water Contact Angle 

The TFN membranes’ hydrophilic characteristics were assessed by measuring alterations in their water contact angles. As demonstrated in Figure 6c, the findings reveal that the unadulterated TFC exhibited a contact angle of 61, whereas the TFN membrane containing 80 ppm of TiO_2_ NSs exhibited the most minimal water contact angle of 42 compared to all other membranes scrutinized. This signifies that the incorporation of TiO_2_ NSs increases the hydrophilic nature of the TFN membrane owing to the hydrophilic attributes of TiO_2_ NSs. The enhancement in hydrophilic properties can be attributed to abundant hydroxyl groups in TiO_2_ NSs incorporated into the PA layer, which can attract water molecules to the surface, thus heightening its hydrophilicity [46,47,48].

### 4.2. Membrane Performance

#### 4.2.1. Salt Rejection

**Figure 7:**
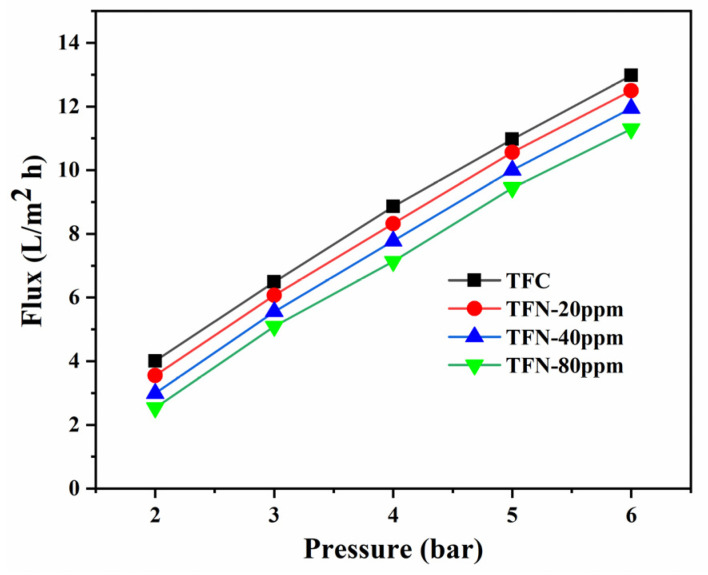
Pure water flux vs. applied pressure.

In the beginning, the pure water that flows through the fresh membrane under operational pressure is utilized to evaluate membrane permeability. All membranes exhibited a proportional increase in flux across the tested range of pressure differentials up to 6 bars, as illustrated in Figure 7.

**Figure 8:**
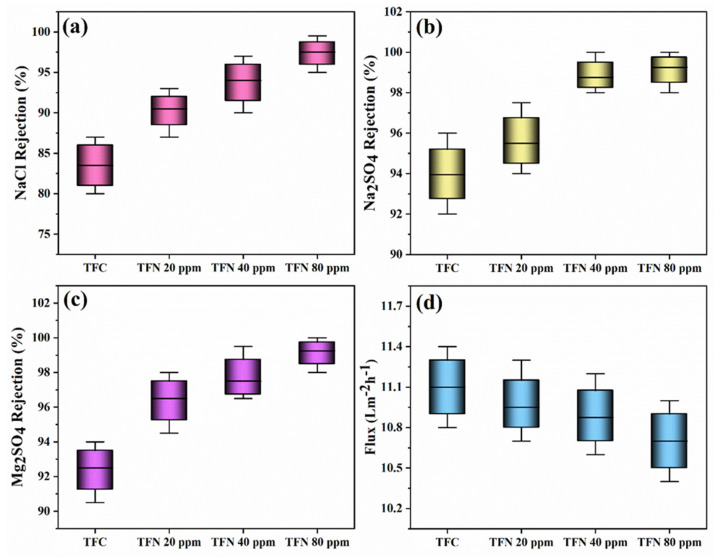
(**a**) NaCl rejection (2000 ppm), (**b**) Na_2_SO_4_ rejection (1000 ppm), (**c**) MgSO_4_ rejection (1000 ppm), and (**d**) flux of TFC and TFN membranes at 5 bar, room temperature, and 1.4 L/min.

The influence of embedding TiO_2_ NSs into the PA layer on the performance of TFN membranes, particularly in terms of flux and salt rejection, has been evaluated through filtration experiments involving NaCl (2000 ppm), Na_2_SO_4_ (1000 ppm), and MgSO_4_ (1000 ppm) solutions, as presented in Figure 8. TFN membranes’ water flux experienced a slight decrease, declining from 11 to 10.6 LMH at 5 bar. In parallel, NaCl rejection increased from 84% to 98%, corresponding to an enhanced incorporation of TiO_2_ ranging from 0 to 80 ppm. The increase in the concentration of TiO_2_ NSs within the thin PA layer may lead to a reduction in the internal free volumes present within the PA film. As a result, this constrains the transportation pathways for water and salt molecules across the TFN membranes [49]. Furthermore, the considerably negative surface charge of TFN membranes, compared to the TFC membrane, would intensify the Donnan effect, thereby enhancing the rejection of NaCl [50]. Meanwhile, the Na_2_SO_4_ and MgSO_4_ rejections in TFC and TFN membranes exhibited a slight difference.

#### 4.2.2. Fluoride Rejection 

The impact of the initial concentration of fluoride, the pH of fluoride solutions, and the quantity of TiO_2_ NSs incorporated into the PA layer were thoroughly assessed to determine the optimal conditions for the TFN membranes. Understanding how these parameters influence the fluoride removal efficiency is crucial for designing an effective and efficient fluoride removal system using TFN membranes.

##### Amount of Incorporated TiO_2_

**Figure 9:**
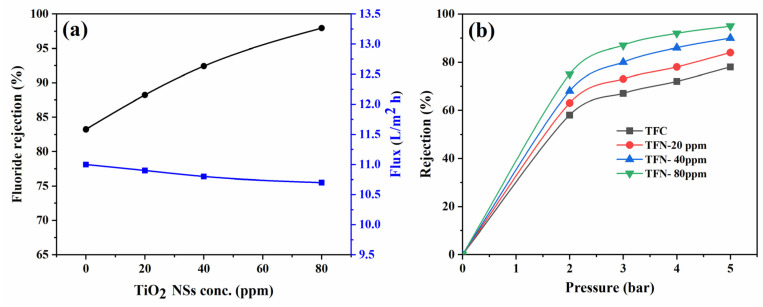
(**a**) Fluoride rejection and flux of TFN membranes as a function of incorporated TiO_2_, and (**b**) fluoride rejection of TFN membranes as applied pressure.

Figure 9 demonstrates that the ability of the TFN membranes to reject fluoride increased with the augmentation of TiO_2_ NSs in the PA layer. Specifically, at a concentration of 80 ppm, the TFN membrane displayed a remarkable fluoride rejection rate of 98% at 500 ppm fluoride concentration and 5 bar applied pressure. In contrast, the TFC membrane exhibited a slightly lower rejection rate of 83%. The enhanced rejection observed in the TFN membrane can be attributed to increased TNS concentration, which may result in a higher degree of crosslinking within the thin PA layer during the polymerization process. Consequently, this crosslinking enhances the resistance of the PA layer to the passage of fluoride ions. Additionally, hydrogen bonding interactions among water molecules and the metal-hydroxyl group on the TiO_2_ NSs surface hinder the direct connection between fluoride ions and the membrane surface, thereby improving the fluoride rejection ability [19]. On the other hand, the surface charge of the TFN membranes plays a significant role in fluoride rejection, where the fluoride rejection of TFN membranes increases with the amount of incorporated TiO_2_ NSs. The F rejections versus pressure for both TFC and TFN membranes are illustrated in Figure 9b. As the pressure increased, F rejections also showed an increment, signifying enhanced filtration as more water permeated through the membrane. This led to a higher dilution of the permeate stream, resulting in a lower permeate concentration.

##### Initial Concentration

**Figure 10:**
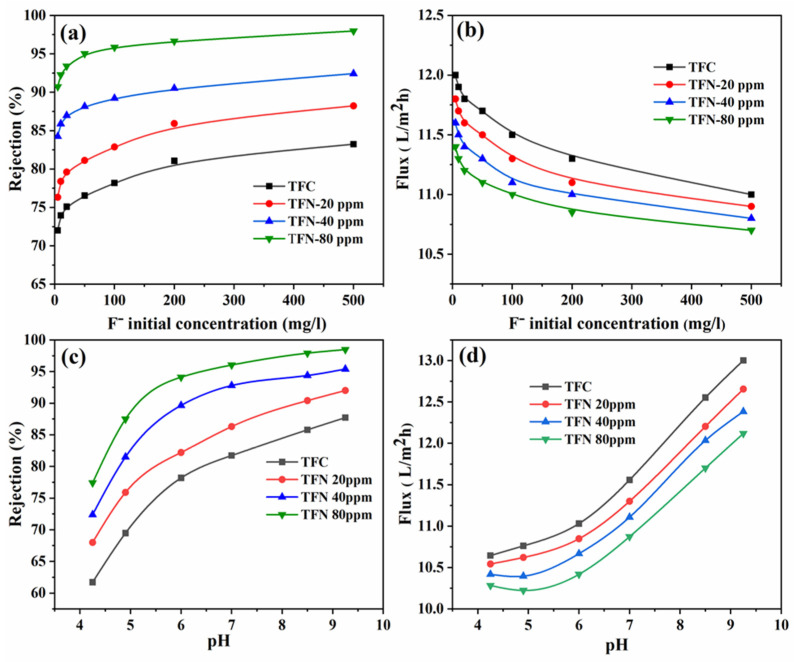
(**a**) The relationship between fluoride rejection and fluoride initial concentrations, (**b**) flux with fluoride initial concentration, (**c**) the relationship between fluoride rejection and pH, and (**d**) flux with pH.

**Figure 11:**
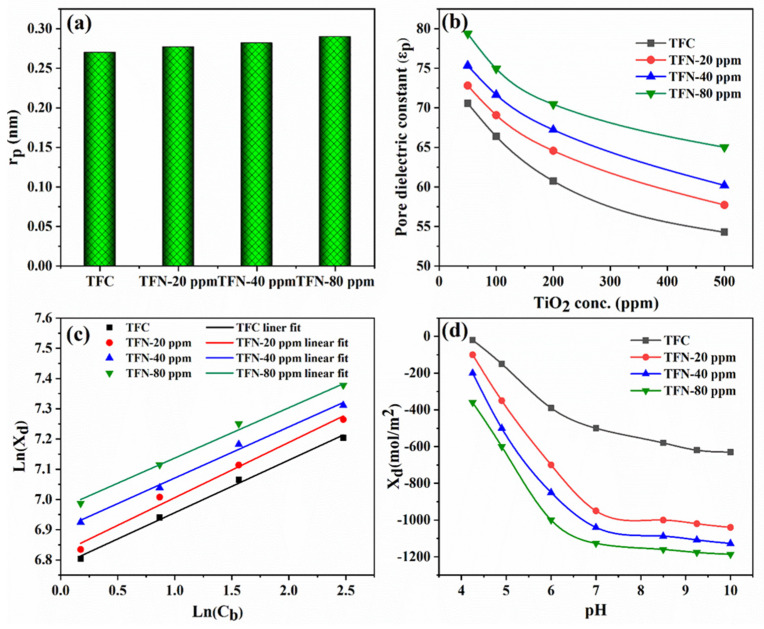
(**a**) Pore radius both of TFC and TFN membrane from the fitting of the DSPM-DE model, (**b**) pore dielectric constant as a function of incorporated TiO_2_, (**c**) charge density as a function of fluoride concentrations corresponding to Freundlich’s adsorption isotherms, and (**d**) membrane charge density vs. pH.

Studying the effect of the initial concentration of fluoride ions in the feed water is crucial for determining the optimum operating range of the fabricated TFN membranes. Figure 10a,b depict the impact of the initial concentration on the fluoride rejection and flux of TFN membranes. The rejection of fluoride grows as the initial concentration of TiO_2_ NSs increases from 5 ppm to 500 ppm, a phenomenon that is probably linked to the influence of the Donnan potential in excluding inorganic solutes [51]. The Donnan potential greatly influences fluoride rejection at low to medium concentrations. However, the Donnan potential at low concentrations becomes insignificant due to the minimal presence of anions and cations [52]. Analyzing the experimental fluoride rejection data of TFN membranes, as presented in Figure 11a, it is evident that, with an increase in fluoride concentration from 5 ppm to 500 ppm, the rejection of TFN membrane increased impressively, reaching 98% at 500 ppm. The high fluoride rejection of the TFN membranes can be attributed to the significantly negative charge of the PA layer, as indicated in the zeta potential results, resulting from the incorporation of TiO_2_ NSs in this layer.

On the other hand, as depicted in Figure 10b, the permeate flux decreased with an increase in feed concentration. This decline could be attributed to fluoride accumulation near the PA layer’s surface, increasing concentration polarization and influencing the permeate flux [8]. Additionally, as the feed concentration increases, the osmotic pressure rises due to the presence of ionic particles. Consequently, the transfer pressure across the membrane decreases, resulting in a decline in permeate flux and fluoride rejection [53].

##### pH of Fluoride Solution 

Figure 10c depicts the relationship between fluoride rejection and pH, a critical factor significantly affecting membrane charge and solution chemistry. The investigation involved a fluoride concentration of 100 mg/L, with pH levels varying from 4 to 9.6. As depicted in Figure 10c, the TFC and TFN membranes demonstrated their lowest rejection of fluoride ions when the pH was close to their respective isoelectric points. This phenomenon can be attributed to the absence of the Donnan effect, with steric size exclusion being the predominant factor influencing fluoride rejection at these pH levels. However, a noteworthy trend emerged as the pH increased from 4.0 to 9.6—the fluoride rejection exhibited a significant rise. For the TFC membrane, fluoride rejection increased from 63% to 87%, while the TFN membrane containing 80 ppm of TiO_2_ NSs rose from 78% to 98%. At pH values exceeding the isoelectric point, the negative surface charge of the membrane increases, as evidenced by the zeta potential findings (refer to Figure 6b), leading to enhanced rejection of fluoride ions. The negative charge could potentially result from the loss of a proton carboxyl from the PA layer under conditions of elevated pH. This particular occurrence leads to an increase in surface charge density, thereby facilitating the phenomenon of Donnan’s exclusion of the fluoride ions [17]. Notably, the TFN membrane containing 80 ppm demonstrated the highest fluoride rejection, confirming the influence of the incorporated TiO_2_ NSs and their negative charge. Furthermore, the flux gradually increased as the feed pH increased, as seen in Figure 10d. This heightened flux can be attributed to the augmented number of negative charges, inducing increased repulsion between polymer segments and consequently expanding the free volume of the PA layer [17]. Furthermore, a high pH reduces solution viscosity, potentially promoting a turbulent flow of the solution over the membrane’s active surface, reducing concentration polarization, and further amplifying permeate flux [22]. 

### 4.3. Fluoride Removal Efficiency 

The developed membrane, which includes TiO_2_ nanosheets, demonstrates an impressive fluoride removal efficiency of about 98%, even when faced with high concentrations of up to 500 ppm and low pressure of 5 bar. To assess its effectiveness, the fluoride removal efficiency of these membranes was compared with commercially available ones mentioned in previous studies (refer to Table 2). These results confirm that the developed membranes can potentially improve fluoride removal technologies, leading to advancements in public health and promoting environmental sustainability.

### 4.4. Donnan-Steric-Pore-Model-Dielectric-Exclusion Model

The dataset of fluoride rejection was analyzed by fitting it to the DSPM-DE model to determine the TFN membranes’ parameter values. This fitting process was conducted using the SDO algorithm. Demonstrated in Figure 11a is the obtained pore radius value from applying the DSMP model to the TFN membrane, which contains 80 ppm TiO_2_ NSs, is 0.27 nm. At the same time, the TFC membrane displays a pore radius of 0.25 nm. These findings suggest that the presence of TiO_2_ NSs had an insignificant influence on the pore radius of the PA layer. Figure 11b exhibits the dielectric constant values obtained through the optimal fitting of fluoride rejection. It is essential to note that, as fluoride concentrations rise, the dielectric constant diminishes due to the existence of ions, leading to increased water confinement within the pores [62]. As the attention of ions within the pores augment, their influence on water molecules strengthens, ultimately resulting in a diminished dielectric constant, which aligns with a corresponding finding mentioned by Micari et al. [63]. The charge density of the TFN membrane can be affected by changes in the feed composition, the pH level, and the composition of the membrane itself. Figure 11b provides a visual representation of the charge density values obtained from applying the DSMP model on the fluoride rejection data of the TFN membranes, which contain various concentrations of incorporated TiO_2_ NSs in the PA layer. The surface charge density is not constant but somewhat greatly dependent on the concentration of fluoride ions. This reliance can be expressed using isotherms that follow a linear pattern A type of isotherm known as Freundlich can also be employed to establish a correlation between the values and the concentrations in the bulk, as demonstrated in the following equation [25]:

$$\left|X_{d}\right|=aC_{b}^{n}$$

A noticeable correlation appears between the fluoride concentration and the surface charge density obtained from fitting rejection data with the DSMP-DE model, as shown in Figure 11c. 

Furthermore, the results derived from Freundlich’s adsorption isotherms validate that the surface charge density in TFC and TFN membranes can be linked to the fluoride concentration level. Figure 11d presents the surface charge density measurements of TFC and TFN membranes obtained by fitting the DSMP-DE model at different pH levels. It is important to emphasize that the charge density values are not constant and exhibit a clear correlation with the pH level. The higher charge density could be attributed to the deprotonation of carboxylic acids within the PA structure at higher pH levels. Consequently, this phenomenon may substantially augment the surface charge density [64].

## 5. Conclusions

This study successfully demonstrated the potential of TFN membranes in addressing the critical issue of fluoride contamination in drinking water. The uniform dispersion and effective incorporation of TiO_2_ NSs were crucial in achieving optimal fluoride removal performance. Incorporating TiO_2_ NSs enhanced the properties of TFN membranes, including surface charge, hydrophilicity, and smoothness, which led to improved fluoride rejection without any negative impact on membrane water flux. The TFN membrane, which had a concentration of 80 ppm of TiO_2_ NSs, exhibited a notable fluoride rejection rate of 98%. Using the DSPM-DE model to fit the experimental data, it was found that the embedded TiO_2_ NSs within the PA layer had a significant effect on the surface characteristics of the TFN membrane. The X_d_ values of TFC and TFN membranes exhibited an upward increase in negativity with an increase in fluoride concentration. This phenomenon was ascribed to the adsorption of fluoride ions into the surface of the membrane. Notably, the TFN membrane’s X_d_ values surpassed those of the TFC membrane, potentially due to the presence of TiO_2_ NSs within the PA layer of the TFN membrane. Enhancing the membrane’s efficiency in removing fluoride is further achieved by optimizing operational parameters, including pH, fluoride concentration, and pressure. The resulting nanocomposite nanofiltration membrane holds great promise for practical application in water treatment systems, offering an advanced and sustainable solution for fluoride remediation. Future research and development efforts should focus on scaling up the production of these membranes, conducting long-term performance evaluations, and exploring potential modifications to improve fluoride removal efficiency further and address real-world water treatment challenges. Overall, this study confirms the capacity of nanotechnology-based strategies to enhance water purification technologies in order to improve public health and promote environmental sustainability.

## Figures and Tables

**Table 1:** Equations for the DSPM-DE model [24,25].

Equations for the Donnan Steric Pore Model with the Dielectric Exclusion (DSPM-DE)	
Expanded Nernst–Planck equation:	
$J_{i}=-D_{i,p}\frac{dc_{i}}{dx}+K_{i,c}c_{i}J_{v}-\frac{Z_{i}c_{i}D_{i,p}F}{RT}\frac{d\psi }{dx}$	(3)
$J_{i}=C_{pi}J_{v}$	(4)
Potential gradient:	
$\frac{d\psi }{dx}=\frac{\sum_{i=1}^{n}\frac{z_{i}J_{v}}{D_{i,p}}(K_{i,c}c_{i}-C_{pi})}{\frac{F}{RT}\sum_{i=1}^{n}\left(z_{i}^{2}c_{i}\right)}$	(5)
Donnan-steric partitioning:	
$\frac{c_{i}}{C_{i}}=\emptyset exp\left(-\frac{z_{i}F}{RT}∆\psi_{D}\right)$	(6)
Hindrance factor:	
$K_{i,d}=1.0-2.30\lambda +1.154\lambda^{2}+0.224\lambda^{3}$	(7)
Steric partition coefficient:	
$\frac{c_{i}}{C_{i}}=\emptyset exp\left(-\frac{z_{i}F}{R.T.}∆\psi_{D}\right)$	(8)
Hindrance factor for diffusion:	
$K_{i,c}=\left(2-\phi \right)(1.0+0.054-0.988\lambda^{2}+0.441\lambda^{3})$	(9)
$\phi ={\left(1-\lambda \right)}^{2}$	(10)
$\lambda =\frac{r_{i}}{r_{p}}$	(11)
Electro-neutrality condition:	
$\sum_{i=1}^{n}z_{i}C_{f,i}=0\;\;\sum_{i=1}^{n}z_{i}C_{p,i}=0$	(12)
Membrane charge density:	
$\sum_{i=1}^{n}z_{i}c_{i}=-X_{d}$	(13)

**Table 2:** Comparative analysis of fluoride removal efficiency between TiO_2_ NS-modified membranes and commercial membranes reported in previous studies.

Membrane Type	Pressure (bar)	Flux LMH	Fluoride mg/L	Removal Efficiency (%)	Ref.
NF400 (PA)	10	----	20	86.1	[54]
Commercial BW30 (Dow FilmTech™, Dayton, OH, USA )	9.8	20	50	80	[55]
BW30 (Dow FilmTech™, Dayton, OH, USA )	6	11.7	56.2	>95	[56]
NF270 (Dow FilmTech™, Dayton, OH, USA )	6	33.5	56.2	54	[56]
NF90 (Dow FilmTech™, Dayton, OH, USA )	15	20.6	17.7	91	[57]
BW30	10	19	212	90.6	[58]
RO Spiral-wound TFC (Vontron, Guiyang, China)	5	----	10	89.81	[59]
NF/RO UTC-60	5	10.9	3	80	[60]
RO membranes (BW30)	10	19	239.9	90.8	[61]
TiO_2_ NSs-incorporated PA TFN membrane	5	10.6	500	98	This work
TiO_2_ NSs-incorporated PA TFN membrane	5	11.7	20	95	This work

## Data Availability

Data are available within the article and Supplementary Materials.

## Supplementary Materials

The following supporting information can be downloaded at: https://www.mdpi.com/article/10.3390/nano14080731/s1, Figure S1. (a) TEM image of the TiO2 NSs and (b) simulated MountainsMap1 software of TEM image of the TiO2 NSs, Figure S2. Magnified HRTEM image of an NSs Reference [65] is cited in Supplementary Materials.

## Nomenclature

A	The effective area of the membrane (m^2^)
$c_{i}$	ion (*i)* concentration within a pore (mol m^−3^)
$C_{i}$	concentration of solute (mol m^−3^)
$C_{f,i}$	solute concentration in feed (mol m^−3^)
$C_{p,i}$	solute concentration in permeate (mol m^−3^)
$D_{i,d}$	coefficient of diffusion in the pore
$D_{i,p}$	hindered diffusivity
$D_{i,\infty }$	diffusion coefficient of species *i* in water (at an infinite dilution)
F	Faraday constant value (96,487 C mol^−1^)
$J_{0}$	flux of permeate (Lm^−2^ h^−1^)
$J_{i}$	mole flux of the solute (mol/m^2^/s)
$J_{v}$	volume flux (m^3^ m^−2^ s^−1^)
$K_{i,c}$	the coefficient of hindrance for convection
$K_{i,d}$	The coefficient of hindrance for diffusion
n	number of markets
N	number of data that has been tested
P	applied pressure (bar)
R	gas constant (8.314 J mol^−1^ K^−1^)
$r_{i}$	radius of solute
$r_{p}$	the radius of effective pore
$R_{exp}$	experimental rejection
$R_{model}$	rejection obtained model
Q	volume of permeated water (L)
$Z_{i}$	valence of ion *i*, dimensionless
T	temperature (*K)*
X_d_	effective charge of the membrane
**Greek letters**	
∆t	permeation time (h)
$∆\psi_{D}$	Donnan potential (V)
$\varepsilon_{p}$	Pore dielectric constant
λ	the ratio of the radius of solute to the radius of effective pore, dimensionless
$\sigma_{F}$	The feed’s ionic conductivity
$\sigma_{P}$	The permeate’s conductivity
∆π	osmotic pressure difference
ϕ	dimensionless steric partition coefficient
ψ	electric potential (V)
∆t	permeation time (h)
$∆\psi_{D}$	Donnan potential (V)
