# Supplementary material for: Fluoride Removal Using Nanofiltration-Ranged Polyamide Thin-Film Nanocomposite Membrane Incorporated Titanium Oxide Nanosheets
Source: Nanomaterials (Basel). 2024 Apr 22;14(8):731. doi: 10.3390/nano14080731 (PMC11053899; doi:10.3390/nano14080731)
Supplement: Supplementary file 1 [file nanomaterials-14-00731-s001.zip › nanomaterials-2933300-supplementary.pdf]

nanomaterials

Supplementary Materials

Fluoride Removal Using Nanofiltration-Ranged Polyamide Thin-Film Nanocomposite Membrane Incorporated Titanium Oxide Nanosheets

Fekri Abdulraqeb Ahmed Ali [1], Javed Alam *[2], Saif M. H. Qaid [3], Arun Kumar Shukla [2], Ahmed S. Al-Fatesh [4], Ahmad M. Alghamdi [1], Farid Fadhillah [1], Ahmed I. Osman *[5] and Mansour Alhoshan *[2,4]

1 Chemical Engineering Department, College of Engineering, Imam Mohammad Ibn Saud Islamic University (IMSIU), Riyadh 11432, Saudi Arabia; feaali@imamu.edu.sa (F.A.A.A); amsalghamdi@imamu.edu.sa (A.M.A); fffadhillah@imamu.edu.sa (F.F)

2 King Abdullah Institute for Nanotechnology, King Saud University, P.O Box- 2455, Riyadh 11451, Saudi Arabia; javaalam@ksu.edu.sa (J. A); ashukla@ksu.edu.sa (A.S).

3 Department of Physics & Astronomy, College of Sciences, King Saud University, P.O. Box 2455, Riyadh 11451, Saudi Arabia; sqaid@ksu.edu.sa (S.M.H.Q)

4 Chemical Engineering Department, College of Engineering, King Saud University, P.O. Box-2455, Riyadh-11451, Saudi Arabia; aalfatesh@ksu.edu.sa (A.S.A-F); mhoshan@ksu.edu.sa (M. A)

5 School of Chemistry and Chemical Engineering, Queen's University Belfast, Belfast, BT9 5AG, Northern Ireland, UK; aosmanahmed01@qub.ac.uk

* Correspondence: javaalam@ksu.edu.sa (J. A), aosmanahmed01@qub.ac.uk (A.I.O) mhoshan@ksu.edu.sa (M. A).

Preparation of TiO_2 Nanosheet

Pure anatase TiO_2 nanoparticles (NPs) were produced by alcoholysis of TNBT alone in ethanol. In contrast, anatase TiO_2 nanosheets (NS) and high-purity brookite TiO_2 nanorods (NRs) required the addition of HF and NaF, respectively. The possible growth mechanism is discussed accordingly. The TiO_2 precursor and a morphology control agent mixture were then placed in an electric oven at 180°C for 24 hours. The atomic ratio of fluorine to titanium (F:Ti) was maintained at 1:1 for the above reaction. After completion of the solvothermal reaction, the autoclave was allowed to cool naturally to room temperature. The white, single-crystalline TiO_2 NS precipitate was collected, washed three times with ethanol and distilled water, and separated by high-speed centrifugation. It was then dried in an oven at 60°C for about 6 hours.

Characterization of Titanium Oxide Nanosheets

The presence of sheet-shaped structures, with an average width falling within the range of 40 to 60 nanometers and a thickness of approximately 7 to 10 nanometers, is evident in the TEM image depicted in Figure S1a. This observation is further supported by a simulated image, generated using the Mountains Map software, as exemplified in Figure S1b. These sheet-shaped structures predominantly constitute around 80% of the {001} outer surface in the nanocrystals. The single crystalline nature of the TiO_2 NSs is evident from the HRTEM image in Figure S2, where the lattice planes can be noticed.

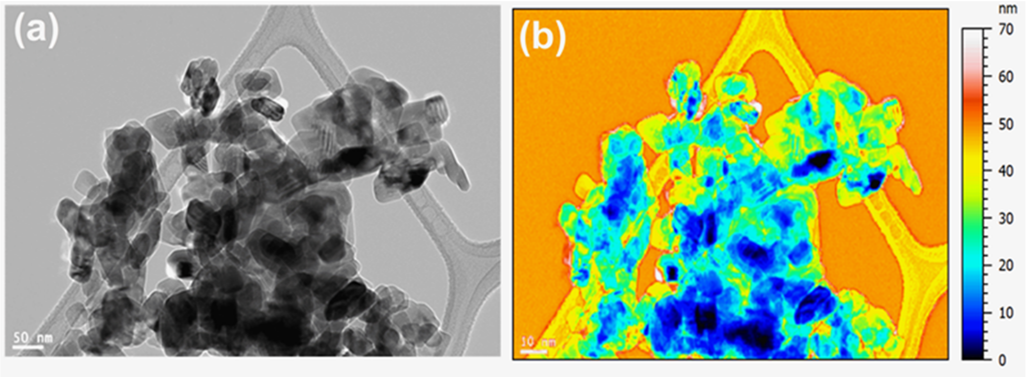

Figure S1. (**a**) TEM image of the TiO_2 NSs and (**b**) simulated MountainsMap1 software of TEM image of the TiO_2 NSs.

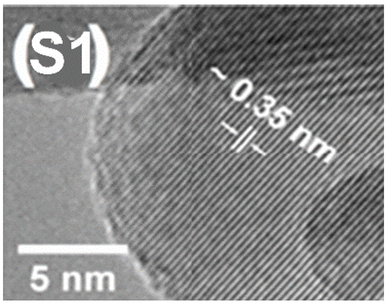

Figure S2. Magnified HRTEM image of an NSs [1].

1. Qaid, S.M.H.; Ghaithan, H.M.; Bawazir, H.S.; Bin Ajaj, A.F.; AlHarbi, K.K.; Aldwayyan, A.S. Successful Growth of TiO2 Nanocrystals with {001} Facets for Solar Cells. *Nanomaterials* **2023**, *13*, 928, doi:10.3390/nano13050928.
